# Fusion of motion smoothing algorithm and motion segmentation algorithm for human animation generation

**DOI:** 10.1371/journal.pone.0318979

**Published:** 2025-02-25

**Authors:** Shinan Ding

**Affiliations:** College of Comic and Animation, Kyungil University, Gyeongsan, Korea; Tongji University, CHINA

## Abstract

In the field of human animation generation, the existing technology is often limited by the dependence on large-scale data sets, and it is difficult to capture subtle dynamic changes when processing motion transitions, resulting in insufficient animation fluency and realism. In order to improve the naturalness and diversity of human animation generation, a method combining motion smoothing algorithm and motion segmentation algorithm is proposed. Firstly, the tree-level model based on human skeleton topology and bidirectional unbiased Kalman filter are used for noise reduction pre-processing of motion data to improve the accuracy of motion capture. Then, combining the discriminant analysis algorithm based on sparse reconstruction and the multi-scale temporal association segmentation algorithm, the key motion segments of the behavior pattern change are identified adaptively. The experimental results show that the accuracy of the proposed algorithm reaches 0.96 in coarse-grained segmentation and 0.91 in fine-grained segmentation, and the segmentation time is 15 seconds on average, which significantly exceeds the prior art. In addition, the algorithm shows superior results in color fidelity, detail representation, motion fluency, frame-to-frame coherence, overall animation consistency, action authenticity, and character expressiveness, and the average user satisfaction is above 0.85. The research not only enhances the naturalness and diversity of human body animation, but also provides a new impetus for technological advances in computer graphics, virtual reality and augmented reality.

## 1. Introduction

Generating natural and realistic human animations is always a hot research topic in computer graphics and animation production. With the virtual reality, augmented reality, and motion capture technologies, the demand for high-quality human animation is increasing [1]. However, existing technologies still face many challenges in generating complex actions and transitional behaviors. For example, data-driven methods often rely on large-scale datasets of specific bone structures, which not only limits the diversity of animations but also increases computational and storage costs [2,3]. In addition, existing algorithms often struggle to capture subtle dynamic changes when handling action transitions, resulting in a lack of smoothness and realism in the generated animation [4,5]. Existing studies mostly focus on the improvement of a single technology or algorithm, lacking systematic research on action data preprocessing and segmentation. Therefore, a method combining motion smoothing and segmentation algorithms is proposed to improve the quality and diversity of human animation generation. The innovation of this study lies in combing a tree-like hierarchical model of human skeleton topology and a clustering algorithm based on sparse representation. This can achieve more accurate segmentation of action sequences and animation generation. A human kinematic model based on bidirectional unbiased Kalman filter is developed. This model can accurately estimate the rotation angle of relevant nodes from raw motion data containing noise, improving the accuracy and reliability of motion capture. The main contributions of the research are as follows: (1) An innovative human body animation generation method was developed, which significantly improved the processing efficiency of 3D human body pose data and the naturalness of animation generation by integrating motion smoothing algorithm and motion segmentation algorithm. (2) The proposed bidirectional unbiased Kalman filter effectively reduced the noise in the motion capture data and improved the accuracy of the motion capture. (3) Discriminant analysis based on Sparse Reconstruction and the application of DASR and Multi-Scale Time Correlation Segmentation (MSTCS) can automatically identify the key motion segments of behavioral pattern change without manually specifying the number of clusters.

In summary, the research not only provides a new perspective in theory, but also provides new tools and methods for the technological development of computer graphics, virtual reality and augmented reality in practice, which is expected to promote further innovation and application expansion in these fields.

## 2. Related works

In recent years, significant research progress has been made in this field, including deep learning based motion synthesis methods, 3D pose parameter capture and adaptation techniques, and coordination methods for virtual scene layout and character motion. To create diverse natural human action videos, Guo et al. designed a system that randomly generated a series of 3D poses that matched specific action categories during the action2motion stage. Subsequently, in the motion2video stage, these 3D poses were further processed and visually rendered, which were ultimately converted into a 2D video format. This method had more advantages in effectiveness than current cutting-edge technologies [6]. Li et al. proposed a generation model based on GANimator to overcome the dependence of existing data-driven motion generation techniques on large and specific bone structure datasets. This model could learn and synthesize new motion patterns based on a single short motion sequence. GANimator only needed to train a single motion sequence to generate new movements suitable for diverse bone structures [7]. Tous et al. used OpenPose and SMPLify-X techniques to analyze the sampled frames in the video, thereby obtaining the 3D pose parameters of all characters in the video. Afterwards, these captured parameters were adapted to a pre-selected 3D model and simulated using cell shadow techniques to create a 2D cartoon visual effect. To generate a continuous smooth animation of the character, interpolation was applied to the gaps between sampled frames. The conclusion confirmed the effectiveness of this method [8]. Zhang et al. utilized neural state machines to achieve coordination and consistency between virtual scene layout and character motion through iterative optimization processes. The collision and interaction between characters and objects were considered. The text content was parsed into a semantic scene graph. Subsequently, 3D object models and motion clips of character animations were retrieved for virtual scene synthesis. The system could generate animation effects that met expectations based on text descriptions [9].

Image recognition and classification also plays a pivotal role in the research of animation generation. Chen et al. proposed to dynamically optimize the rank diagonal elements of the representation matrix for efficient representation and classification of images. This could enhance the distinction between analytical and synthetic dictionaries, while clearing non-diagonal block elements to zero. This method could at least match or even surpass existing technologies in image recognition performance. In addition, this method significantly reduced the training and testing time, thus verifying its effectiveness [10]. Sima et al. developed a multi-core mutual learning strategy based on combined mid-level feature transfer learning for classification of hyperspectral images. Their method used a joint sparse representation model to obtain sparse reconstructed features under triple-scale superpixel boundaries and region constraints. This method had significant performance advantages compared to several advanced algorithms based on deep learning [11]. There is a balance issue between accuracy, inference speed, and model complexity in real-time semantic segmentation applications. Gao et al. proposed an innovative lightweight network architecture that utilized multi-scale context fusion technology and explored an asymmetric encoder-decoder structure to improve performance and reduce model size. This architecture only used 1.15M parameters and achieved an average IoU of 71.9% on the Cityscapes dataset, while running at a rate of over 50 FPS on a Titan XP GPU [12]. The encoder-decoder convolutional neural network had difficulty improving accuracy in CT medical image segmentation due to the loss of details during the encoding stage. Regarding this, Xia et al. introduced the Multi-scale Context Attention Network (MC-Net), whose core concept was to mine effective information from different scales and contexts to more accurately segment target objects in medical images. MC-Net outperformed current advanced methods in evaluation metrics such as accuracy, sensitivity, area under receiver operation characteristic curve, and Dice coefficient [13].

In response to the question of how to combine algorithmic animation with augmented reality technology to enhance the learning experience, Paredes-Velasco et al. proposed an architectural approach that allows the generation of interactive algorithmic animation and its integration into an immersive augmented reality environment. The results showed that through the combined use of augmented reality and visualization, students experienced a significant increase in positive emotions during the experience. In addition, face-to-face and online learning modes have significant effects on emotions and learning effects of augmented reality [14]. In order to improve the convenience of human-computer interaction, Wang et al. proposed a deep learn-based expression dynamic capture and 3D animation generation method. For facial expression dynamic capture, a facial feature extraction algorithm based on deep learning was proposed, and support vector machine was used to classify the features. For 3D animation, C++ and OpenGL were used for rendering simulation. The research results showed that the proposed face detection algorithm performed well in both accuracy and speed, and could realize real-time detection of the face region in video images [15]. To solve the problem of insufficient interaction of virtual characters in film and television animation works, Cai et al. proposed a sensor-based human-computer interaction system, which realized the recognition of human body parts and the determination of actions through the algorithm of separating human body from the background environment in the depth image. The research results showed that the efficiency of the system in recognizing the movements of animated characters reached 80%, and it could quickly and accurately capture the body movements of animated characters [16].

To sum up, human animation generation, as a hot topic in the field of computer graphics and animation, has attracted a lot of research attention. From data-driven approaches to the application of deep learning techniques, to the capture and adaptation of 3D pose parameters, researchers continue to explore how to generate more natural and realistic human animation. For example, Guo et al. demonstrated an advantage in effect by systematically generating 3D poses randomly and further processing them into 2D video formats. The GANimator model proposed by Li et al. could learn and synthesize new motion patterns based on a single short motion sequence, reducing the dependence on large-scale data sets. However, existing technologies still face challenges in handling motion transitions, especially when it comes to capturing subtle dynamic changes and generating smooth animations. Most of the existing researches focus on the improvement of a single technology or algorithm, and there is a lack of systematic research on the preprocessing and segmentation of motion data. In addition, it is often difficult for existing algorithms to capture subtle changes in motion when dealing with transitional behavior, resulting in insufficient animation fluency and realism. To solve these problems, an innovative method combining motion smoothing algorithm and motion segmentation algorithm is proposed. The segmentation accuracy of action sequences and the naturalness, fluency and diversity of animation are improved by combining the tree hierarchy model of human skeleton topology and the clustering algorithm based on sparse representation.

## 3. Methods

Existing technologies face significant challenges in handling complex movements and motion transitions, especially in capturing subtle dynamic changes in motion to generate smooth, realistic animations. These problems are mainly due to the dependence of existing algorithms on large-scale data sets of specific bone structures, and the limitations of processing transitional behaviors, resulting in the lack of diversity and realism of animation. In order to solve these problems, a comprehensive method combining motion smoothing algorithm and motion segmentation algorithm is proposed in this paper. Through more accurate preprocessing and segmentation of human motion data, more natural, smooth and diversified human animation is generated.

### 3.1. Preprocessing of human motion animation data based on motion smoothing algorithm

**Fig 1:**
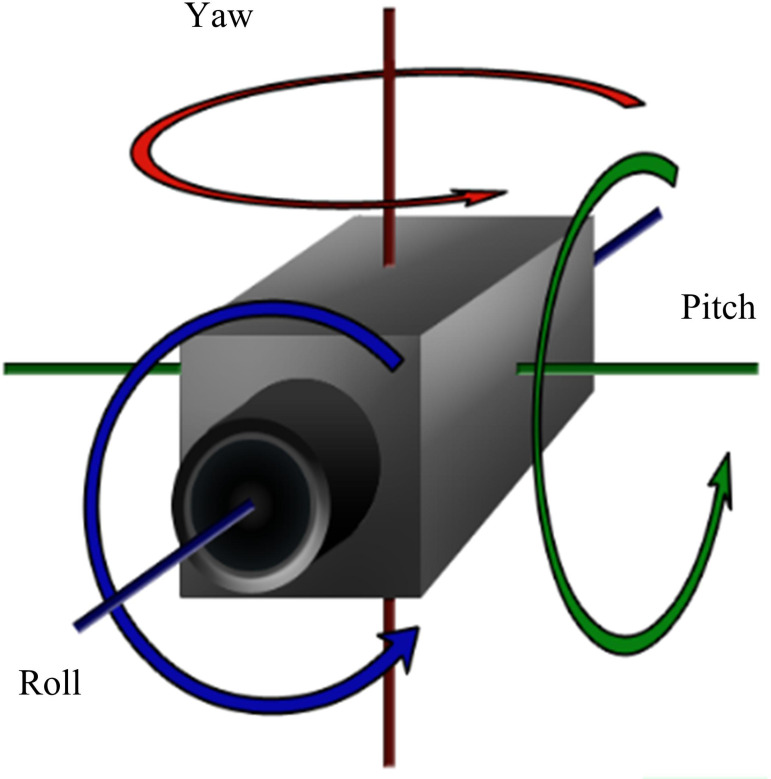
Rotation information in the coordinate system.

Human motion capture technology provides real-world motion data for computer-generated dynamic characters. With the increasing demand for action analysis and action generation applications, the standards for the quality of action data are also becoming higher. However, due to errors in mechanical measurement, instability in system output, and external environmental interference, the data collected by state-of-the-art motion capture systems are inevitably affected by noise interference [17–19]. It is crucial to obtain noise free and pure action data to achieve precise action analysis. Therefore, denoising action data is an indispensable step. The three-dimensional coordinates record each joint point’s position information in the world coordinate system. The rotation angle and quaternion record the rotation information of the joint point in the local coordinate system in Fig 1.

**Fig 2:**
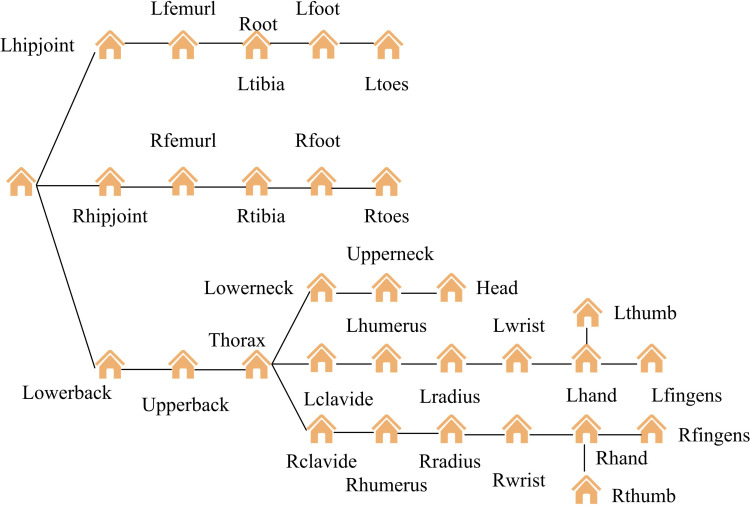
Human skeleton tree hierarchy model.

In most cases, in Fig 1, the changes in the position of the joints in space can intuitively reflect the changes in the human body’s motion state. However, if only three-dimensional coordinates are used to describe human posture, this description method lacks hierarchy. Because the position information of each joint point in the posture description is independent of each other, any change in the position of a joint will not affect the position of other joint points. This method violates the principle of bone length invariance in the human body during filtering processing [20,21]. To satisfy the constraint of keeping the bone length constant while filtering, a tree-like hierarchical model based on the topological structure of the human skeleton is adopted. A hierarchical tree model is established based on the topological structure of the human skeleton in Fig 2.

In Fig 2, tree’s root node is defined as “root”. The root position in the world coordinate system establishes the spatial position of the human pose, within the hierarchical tree model under discussion. Meanwhile, the rotation information of root defines the orientation of human posture. In addition, all other nodes in the model are rotated and positioned in a coordinate system relative to their parent node. In this hierarchical model framework, root node’s global coordinate position at a specific time point is given. Non-root nodes’ global coordinates at the same time point can be derived using corresponding mathematical formulas [22] in equation (1).


$$P_{K}(t)=P_{root}(t)R_{root}(t)T_{root}(t)R_{1}(t)\cdots R_{K-1}(t)T_{K}(t),(K=1,2,\cdots ,M)$$ (1)

In equation (1), $T_{K}(t)$ represents the offset vector from the parent node to the child node. $P_{root}(t)$ represents the global coordinates of the root node at *t*. $P_{K}(t)$ F represents the global coordinates of the joint point *K* at *t*. $R_{K_{i}}(t)$ represents the rotation vector of the joint point $K_{i}$ at *t*. *M* represents the quantity of non-root joint points. In the proposed model, equation (1) not only ensures the constancy of bone length, but also allows for the transformation between the rotation angle of joint points and their coordinates in three-dimensional space. Based on this, a human kinematic model based on a bidirectional unbiased Kalman filter is developed. This model sets the rotation angle of the joint point as a state variable and takes the three-dimensional coordinates of the joint point as an observation variable. The purpose is to accurately estimate the rotation angle of the relevant nodes from the raw motion data containing noise [23]. This bidirectional unbiased Kalman filter includes forward and backward filtering. In the definition of a state vector, it is composed of the rotation vectors of all joint points at a specific time *t*, represented by equation (2).


$$x(t)=(R_{root}(t),R_{1}(t),R_{2}(t),\cdots ,R_{M}(t))$$ (2)

In equation (2), x(t) represents the state vector. Through this method, both the dependency relationship between joint points and the invariance of bone length can be considered in the filtering process. In this paper, a tree hierarchical model and bidirectional unbiased Kalman filter based on human skeleton topology are proposed to deal with these constraints through a series of built-in constraints. The model ensures the invariance of bone length, while taking into account the rotation limitations and dependencies of joints, as well as the effect of hierarchy on movement. In addition, by introducing a noise term in the filtering process, the model can simulate and reduce uncertainties and interference in practical applications, improving the accuracy of the data. In particular, the algorithm can adaptively adjust the segmentation threshold to deal with the subtle dynamic changes during the action transition. By identifying the key motion segments, it generates smooth and realistic human animation. The observation vector is composed of the global coordinates of all joint points at a specific time *t*, represented by equation (3).


$$y(t)=(P_{root}(t),P_{1}(t),P_{2}(t),\cdots ,P_{M}(t))$$ (3)

In equation (3), y(t) represents the state vector. To adapt to diverse types of motion, this study uses a random walk method to simulate state changes. Using equation (1), the motion path of the root node is input. Based on the skeletal structure diagram of the human body shown in Fig 2, the observation paths of all joint points are calculated sequentially. On this basis, during the forward filtering process, the state transition function is represented by equation (4).


x(t)=x(t−1)+η(t) (4)

In equation (4), η(t) refers to the introduction of additional noise terms in describing the state transition function to simulate uncertainty and interference in practical applications. The system observation function is represented by equation (5).


y(t)=H(x(t))+ξ(t) (5)

In equation (5), ξ(t) refers to the introduction of additional noise terms in describing the system observation function to simulate uncertainty and interference in practical applications. H(x(t)) represents the forward kinematics transformation function. In reverse filtering, the state transition and observation function of the system are represented by equation (6).


$$\{\begin{array}{c} x(t)=x(t-1)+\eta^{\prime }(t)\\ y(t)=H(x(t))+\xi (t) \end{array}$$ (6)

In equation (6), $\eta^{\prime }(t)$ indicates that additional random interference terms are added to the system observation model to simulate the noise effects that may be encountered in actual observations.

### 3.2. Coarse-grained motion segmentation based on time-domain analysis

**Fig 3:**
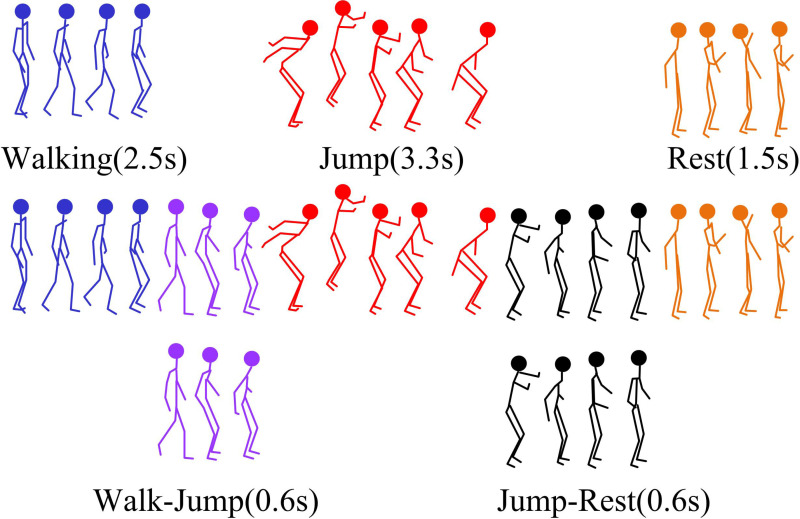
Coarse-grained motion segmentation process.

After preprocessing human motion animation data based on motion smoothing algorithms, this study analyzes the behavioral patterns in motion sequences based on sparse representation. This can overcome the limitations of traditional clustering methods that require manual clustering settings. Traditional clustering algorithms are prone to getting stuck in local optima and are difficult to handle transitional behavior fragments. Subsequently, this study combines contextual information of sequences and multi-scale candidate segmentation schemes to adaptively adjust segmentation thresholds. This effectively identifies key motion segments for behavior pattern transitions from the original sequence [24,25]. Fig 3 shows the coarse-grained motion segmentation process.

**Fig 4:**
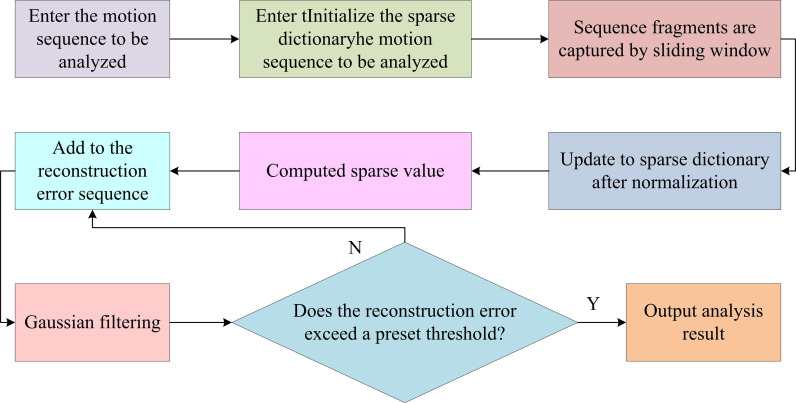
DASR algorithm flow chart.

In Fig 3, the proposed coarse-grained motion segmentation method differs from conventional methods. It focuses on identifying key time nodes where behavioral patterns change from the original complex motion sequence. Based on these time nodes, the original complex motion sequence is divided into several independent single behavior segments and transitional behavior segments. To accurately identify the key time points of behavior pattern transformation in sequences, it should deeply understand the behavioral meanings contained in high-dimensional motion data. The existing algorithms usually use clustering methods to divide the original sequence into multiple independent segments. Each fragment corresponds to a specific category of behavior [26,27]. However, these algorithms generally have a flaw, which is the need to manually specify the quantity of clusters. This quantity will directly affect the accuracy of the final segmentation result. A DASR is proposed to address this issue, with specific steps in Fig 4.

In Fig 4, this method detects the appearance of new behavior types by scanning the original motion sequence point by point and utilizing the changes in sparse reconstruction errors. Once a new type of behavior is discovered, it is added to the existing dictionary and scanning continues. This process will be repeated until it reaches the end of the sequence to determine the behavioral patterns contained in the original complex motion sequence. To address the problem of local optimal solutions in motion sequences, a new algorithm called MSTCS is proposed. This algorithm adopts a dynamic programming strategy to split the minimization of overall clustering costs into multiple local multi-scale clustering cost minimization problems with the same properties. In forward search, multiple candidate solutions with different segmentation scales are reserved for each frame. When backtracking, the optimal segmentation scale is selected for the current frame based on the temporal correlation strategy. The candidate solution corresponding to this scale is used as the optimal segmentation solution for the current frame. MSTCS can adaptively adjust the segmentation scale by combining contextual information of sequences and candidate solutions of different scales. This can effectively identify the key motion segments with behavior pattern changes in the original sequence, solving the difficulty of segmenting local optimal solutions and transitional behavior segments [24,28]. When conducting clustering analysis, it is necessary to comprehensively consider the clustering cost of the entire dataset, represented by equation (7).


$$\{\begin{array}{c} J_{MSTCS}(G,s)=\sum_{c=1}^{k}\sum_{i=1}^{m}g_{ci}||\psi (x_{\left[s_{i},s_{i+1}\right)})-i_{c}|{|}^{2}=||[\psi (Y_{1}),...,\psi (Y_{m})]-ZG|{|}_{F}^{2}\\ s.t.G^{T}I_{k}=I_{m}ands_{i+1}-s_{i}\in [1,n_{\max}],n_{\max}\in \left\{n_{1},n_{2},...,n_{p}\right\} \end{array}$$ (7)

In equation (7), $J_{MSTCS}(G,s)$ represents a cost function. *G* represents a fragment class identification matrix. *k* represents the behavior types contained in the sequence. $x_{\left[s_{i},s_{i+1}\right)}$ represents a motion segment. $s_{i}$ represents the *i* th segmentation point. *m* represents the total segmentation points presented in the sequence. $\psi (Y_{1})$ represents the kernel function. The motion segment is represented by equation (8).


$$x_{\left[s_{i},s_{i+1}\right)}=Y_{i}=\left\{x_{s_{i}},x_{s_{i+1}},...,x_{s_{i+1}-1}\right\}$$ (8)

In equation (8), $Y_{i}$ also represents a sports segment. However, the goal of minimizing the clustering cost of the entire dataset is essentially to find the optimal solution for variables *G* and *i*. This process is an NP-Hard problem. To better illustrate the relationship between global clustering cost and sequence end frame *v*, equation (7) can be rephrased, represented by equation (9).


$$J(v)=\underset{v-n_{\max}\leq i<v}{\min}(J(i-1)+\min\sum_{c=1}^{k}g_{c}h_{\Psi }^{2}(X_{\left[i,v\right]},i_{c}))$$ (9)

**Fig 5:**
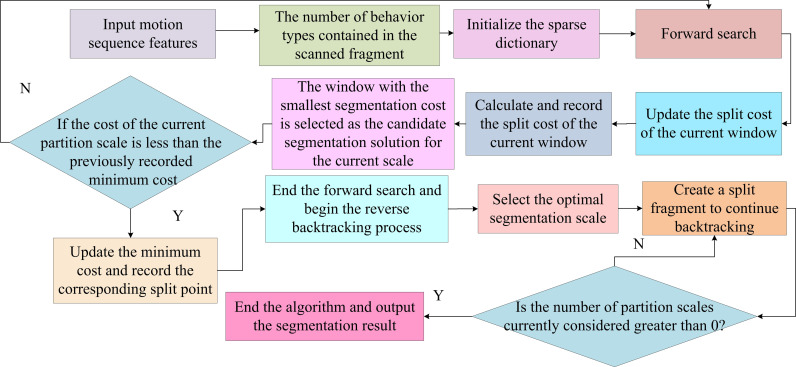
Flow chart of multi-scale time correlation segmentation.

In equation (9), *X* represents the moving segment, *k* represents the number of behavior types contained in the sequence, and *v* represents the end frame of the sequence. $h_{\Psi }^{2}(X_{\left[i,v\right]},i_{c})$ represents two different motion sequences. Through nonlinear kernel functions, these motion sequences are mapped to a high-dimensional space. The square of the distance between them is used to calculate the clustering cost. J(v) represents the clustering cost of a certain segment of motion sequence when describing clustering analysis of motion sequences. The entire motion segment achieves global optimal segmentation only when both adjacent segments have selected their respective optimal local segmentation schemes, and the combined local clustering costs hit their lowest possible sum. When the variable *v* is equal to a specific value *n*, this corresponds exactly to the global minimum clustering cost of the motion sequence. Fig 5 shows MSTCS.

In Fig 5, MSTCS evaluates the segmentation cost for each possible segmentation scale in forward search and selects the optimal segmentation point in backward backtracking, thereby achieving effective segmentation of motion sequences. By considering multi-scale information and temporal correlations, this algorithm can more accurately identify the transition points of behavioral patterns.

### 3.3. Fine-grained motion segmentation based on frequency domain analysis

**Fig 6:**
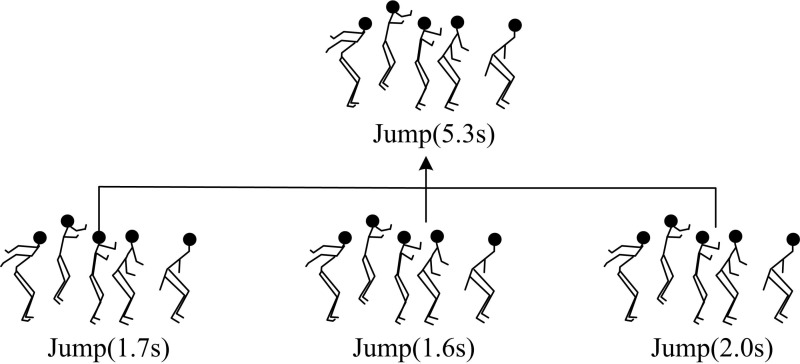
Fine-grained motion segmentation.

In the previous section, by applying DASR and MSTCS, the original complex motion sequence is divided into multiple independent behavior segments and transitional behavior segments, achieving rough segmentation of the motion sequence. To achieve more detailed segmentation results on this basis, the study further subdivides each independent behavior segment obtained after rough segmentation in Fig 6.

In Fig 6, fine-grained motion segmentation refers to the process of identifying key time nodes that transition between different sub periods within a single behavioral segment and dividing the original sequence into multiple periodic segments based on these nodes. In signal processing, the moment when the maximum value of particle vibration velocity occurs is often defined as the periodic segmentation point of a single particle motion signal. The main frequency of the signal corresponds to the frequency at the peak moment of the vibration velocity. Therefore, the main frequency of a signal can largely reflect its periodic characteristics [29,30]. Before performing fine-grained segmentation, frequency domain analysis is first performed on each independent behavior segment. The main frequency of specific behavior segments is calculated to eliminate the adverse effects of noise and intermittent stops on subsequent segmentation. At the dominant frequency, the motion parameters of several major joint points with the highest response values are selected to jointly form the characteristics that express human posture. Meanwhile, the interference of motion parameters of secondary joints on subsequent segmentation is excluded. Considering that the motion states of multiple joints in the human skeleton cannot be completely synchronized in practical situations, this study conducts frequency domain analysis on the motion parameters of each joint separately. The frequency corresponding to the maximum sum of squared frequency responses is used as the main frequency of the segment to be segmented. The rotation angle of each joint point is used as the motion parameter. Fourier transform is applied to the motion parameters of each joint point. After amplitude normalization, the corresponding frequency response is obtained to determine the dominant frequency of the motion segment, represented by equation (10).


$$\omega_{p}=\underset{\omega }{\arg}\max\sum_{i=1}^{M_{total}}||{\hat{f}}_{i}(\omega )|{|}^{2}$$ (10)

In equation (10), $M_{total}$ represents the total motion parameters for all joint points. To minimize the impact of non-critical motion parameters on subsequent segmentation work, the study selects the motion parameters of the first *M* joint points with the highest response peak as the main feature parameters. These main characteristic parameters are combined to form a comprehensive indicator system that can describe changes in human posture, represented by equation (11).


$$\{\begin{array}{c} M=\underset{M}{\arg}\max\sum_{j=1}^{M}||{\hat{f}}_{j}(\omega_{p})|{|}^{2}\\ s.t.\frac{\sum_{j=1}^{M}||{\hat{f}}_{j}(\omega_{p})|{|}^{2}}{\sum_{i=1}^{M_{total}}||{\hat{f}}_{i}(\omega )|{|}^{2}}\geq \theta \end{array}$$ (11)

**Fig 7:**
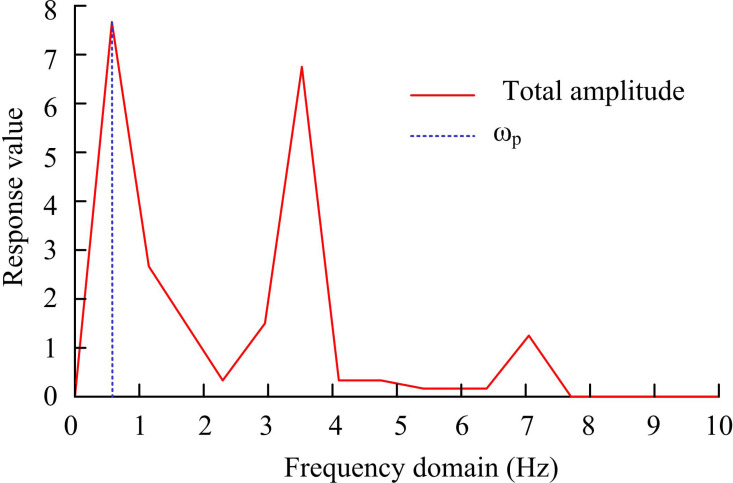
Frequency domain of the motion parameters of the node.

In equation (11), *θ* represents a threshold. Given that human motion typically exhibits symmetry, this study only conducts frequency domain analysis on the joint motion parameters of the left limb, including the left arm and left leg in Fig 7.

In Fig 7, taking frequency as the X-axis and accumulating the frequency responses of all joint motion parameters as the Y-axis, the overall amplitude frequency characteristic curve of the motion segment is plotted. By analyzing the amplitude frequency characteristic curve, the main frequency components in motion can be identified, corresponding to specific motion patterns or behaviors. There may be multiple brief pauses when transitioning between different stages of the motion segment cycle. These pause frames are often identified as potential segmentation points by zero velocity crossing point detection algorithms. If these potential segmentation points are directly used for fine-grained segmentation, it may lead to over segmentation. To address excessive segmentation, traditional methods use clustering algorithms to cluster these potential segmentation points into multiple categories and select one of these categories as the actual segmentation point. However, in practical situations, the quantity of clusters is often difficult to determine in advance. In response to this challenge, an Adaptive K-means (AKM) is proposed, specifically designed to handle the clustering of candidate segmentation points. The clustering cost is composed of a linear combination of intra class distance and inter class distance. This study will transform determining the optimal number of clusters into seeking to minimize clustering costs [31,32], represented by equation (12).


$$K^{*}=\arg\underset{K}{\min}[J(K)]=\arg\underset{K}{\min}[J_{\min}(K)-\lambda *J_{\mathrm{int}er}(K)]$$ (12)

In equation (12), $K^{*}$ represents finding the optimal number of clusters. *λ* is a weight coefficient. $J_{\min}(K)$ represents the minimum clustering cost at the candidate segmentation point *K*. $J_{\mathrm{int}er}(K)$ represents the in-class distance at the split point *K*, which is the sum of the distances between data points within the same class. This study uses an empirical value $N/K^{2}$. $K^{*}$ generally does not exceed 10. The clustering cost function is in most cases a convex function. Therefore, the problem of minimizing clustering costs can be solved through iterative search. *K* is set to 2 during initialization. Then, in each iteration, *K* increases by 1 until the clustering cost reaches its minimum value. To sum up, the bidirectional unbiased Kalman filter needs to update the state of all nodes at each time step, and the computation is relatively large due to the need for forward and reverse filtering at the same time. DASR algorithm needs to process each data point, and the computational complexity is proportional to the length of the sequence. MSTCS algorithm needs to be calculated on multiple scales, which increases the complexity of calculation. AKM algorithm needs to calculate the clustering cost at each segmentation point and select the optimal number of clusters. In fine-grained motion segmentation, it is necessary to perform frequency domain analysis for each independent behavior segment and calculate the main frequency of a specific behavior segment. The proposed methods may be high in computational complexity, especially when dealing with large data sets and multi-scale analysis. If the advantages in segmentation accuracy and animation generation effect are significant, then these computational costs are acceptable in practical applications.

## 4. Results

Firstly, this study evaluated the preprocessing filtering effect of human motion animation data based on motion smoothing algorithms and analyzed the reconstruction error obtained from DASR scanning motion sequences. Next, a performance comparison analysis was conducted on the proposed MSTCS-AKMs, including segmentation accuracy, robustness to noise, and time complexity. Finally, a comparative analysis was conducted on human animation generation effects using different methods.

### 4.1. Analysis of filtering effect and reconstruction error sequence

**Fig 8:**
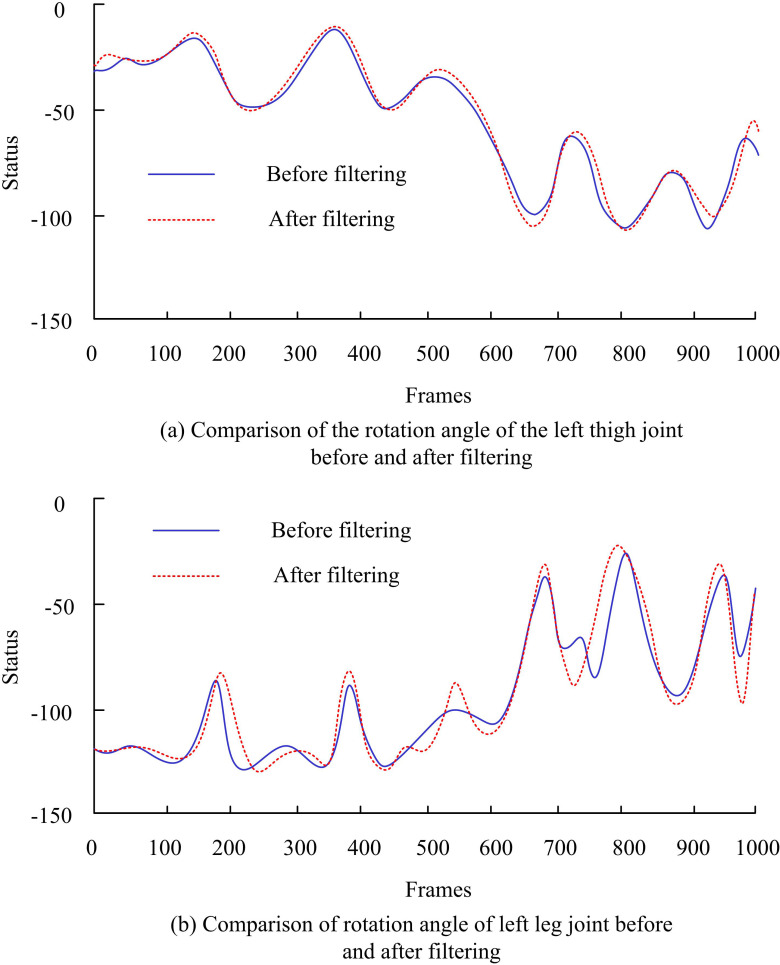
The dynamic curve of rotation angle of each node of human body.

This study selected 14 complex and long-lasting motion sequences from individuals numbered 86 in the CMU exercise database as the analysis objects. These sequences include various independent behavioral patterns, such as walking, running, jumping, boxing, and kicking. Meanwhile, they include some transitional behavior patterns, such as transitioning from walking to jumping, transitioning from jumping to walking, transitioning from walking to running, and transitioning from running to kicking. On average, each sequence contains approximately 8000 frames of images, which are used for motion segmentation research. In order to quantitatively evaluate the accuracy of the proposed MSTS-AKM algorithm in human motion segmentation, a variety of standard evaluation indexes were used. The segmentation accuracy rate is the ratio of the segmentation behavior segment to the total segment number. The average time of segmentation operation was recorded, which reflects the time efficiency of the algorithm. When the segmentation operation was performed, the average utilization of the GPU was monitored, providing the degree to which the algorithm was optimized for parallel computing or GPU acceleration. The effects of human body animation generated by different methods were compared and analyzed, including color fidelity, detail expression, motion fluency, frame to frame coherence, overall animation consistency, motion authenticity, and character expressiveness. Through user survey, the user satisfaction of human animation generated by MSTS-AKM algorithm was evaluated. Before and after filtering, the dynamic curves of the rotation angles of various joints in the human body over time were compared and analyzed in Fig 8.

**Fig 9:**
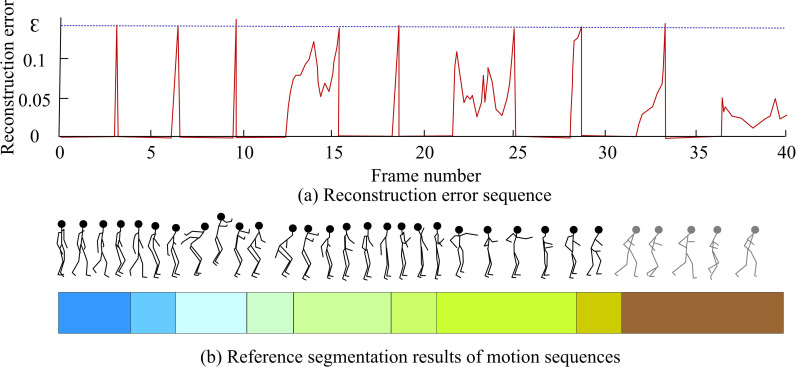
Analysis results of complex motion sequences.

In the CMU exercise database, Fig 8 (a) shows a comparison of the rotation angle change curves of the left thigh joint point of the human body before and after filtering. Fig 8 (b) shows the corresponding comparison of the left calf joint point. For unfiltered raw motion data, there were small fluctuations in the rotation angle curve of the joint points, indicating a low level of noise contained in the raw data. After filtering, most of the original small fluctuations on the curve disappeared, making the overall curve smoother. Fig 9 shows a complex motion sequence containing 9 types of behavior.

Fig 9 (a) shows the reconstruction error curve obtained by scanning the motion sequence with DASR. ε represents the reconstruction error threshold. In Fig 9 (b), the results of human segmentation are identified as independent behavior types through different solid colors. Transition behaviors are represented by different gradient colors. This study set a reconstruction error threshold of 0.14 as the basis for determining the starting frame of a new behavior type. When the window size was set to 300, the effect of sparse discriminant analysis was most ideal. Compared with the results of manual segmentation, the quantity of points where the reconstruction error curve intersected with the horizontal axis was consistent with the behavior types included in the motion sequence. However, the transition point of the reconstructed error curve and the actual segmentation point had a significant deviation. Therefore, DASR technology was only applied to analyze the total behavior types contained in the original sequence, without using it as a basis for precise segmentation.

### 4.2. Comparison of algorithmic performance

**Fig 10:**
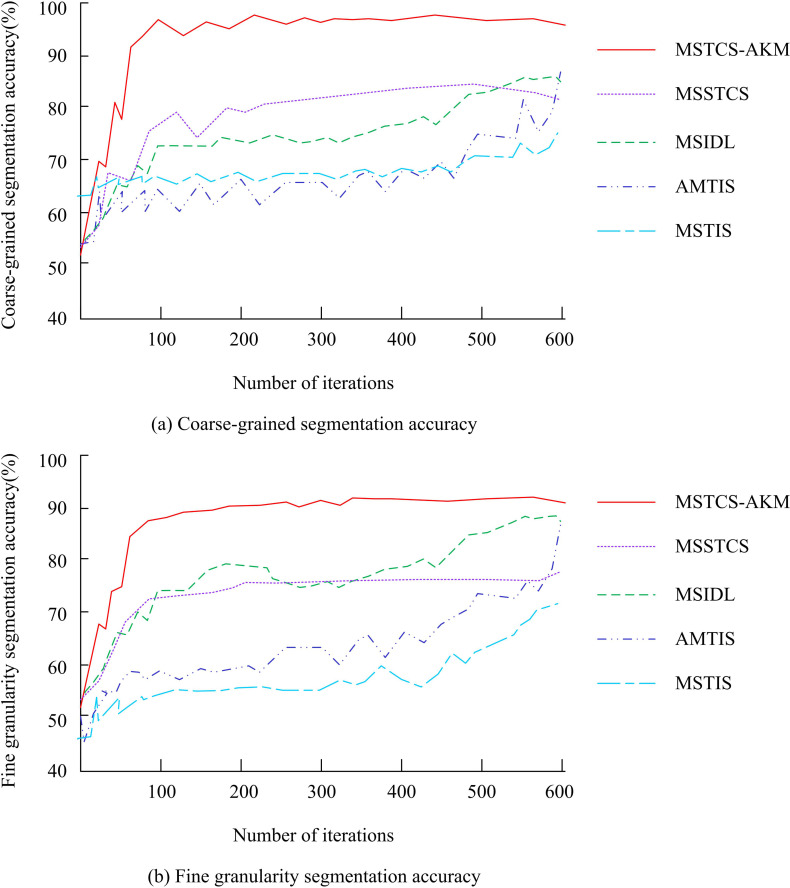
Comparison of segmentation accuracy of different segmentation algorithms.

For the sake of comparison, a comprehensive comparative analysis was conducted on the segmentation results of coarse and fine granularity. This study selected the CMU motion database as a test dataset to comprehensively evaluate the MSTCS-AKM with segmentation accuracy, robustness to noise, and time complexity. Experimental comparative analysis was conducted with four existing algorithms. Comparative algorithms include Multi-Scale Space-Time Image Segmentation (MSTIS), Adaptive Multi-Scale Thresholding Image Segmentation (AMTIS), Multi-Scale Image Segmentation with Spatio-Temporal Context (MSSTCS), and Multi-Scale Image Segmentation with Deep Learning (MSIDL). Fig 10 shows the segmentation accuracy of different segmentation algorithms.

**Fig 11:**
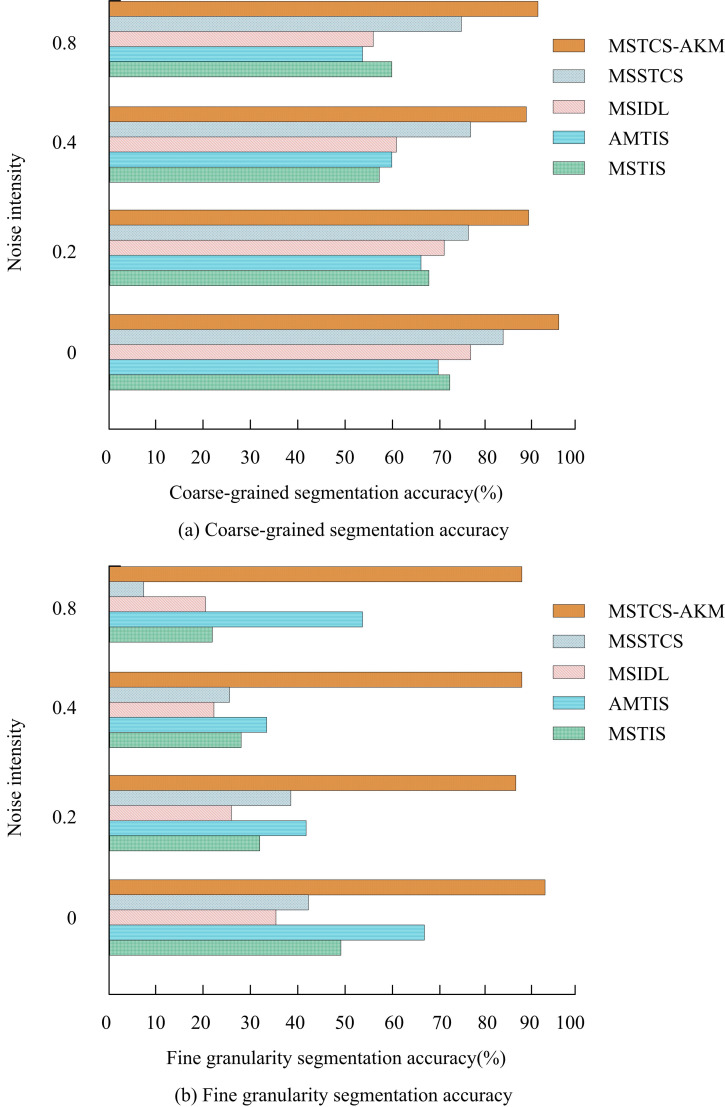
Comparison of segmentation accuracy of sequences with and without noise.

In Fig 10 (a), the accuracy of MSTS-AKM in coarse-grained segmentation ultimately stabilized at 0.96, which was superior to other algorithms. In Fig 10 (b), the accuracy of MSTS-AKM in fine-grained segmentation ultimately stabilized at 0.91, significantly surpassing all other algorithms. MSTS-AKM successfully divided images or videos into smaller and more specific regions, which accurately identified the categories of each region. To demonstrate the advantages of MSTS-AKM in resisting noise interference compared to other segmentation algorithms, Gaussian noise with different variances was artificially added to the original motion data. The accuracy of motion sequence segmentation before and after adding noise was compared. Fig 11 shows the sequence’s segmentation accuracy with and without noise.

**Fig 12:**
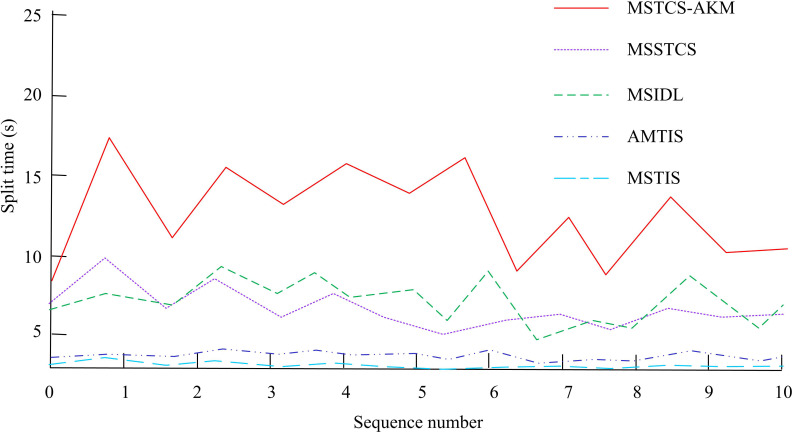
Comparative analysis of segmentation time.

In Figs 11 (a) and (b), after introducing different levels of noise, the accuracy of coarse-grained and fine-grained segmentation in MSSTCS, MSIDL, AMTIS, and MSTIS significantly decreased, with a maximum decrease rate of 85%. In contrast, the accuracy of MSTS-AKM decreased slightly, indicating its stronger robustness to noise. On a system equipped with the fourth generation Intel Core i7-4790K quad core processor, single threaded Matlab programming was used to perform segmentation operations on each set of motion sequences. Fig 12 shows the segmentation time of 10 sets of sequences using different segmentation algorithms.

**Fig 13:**
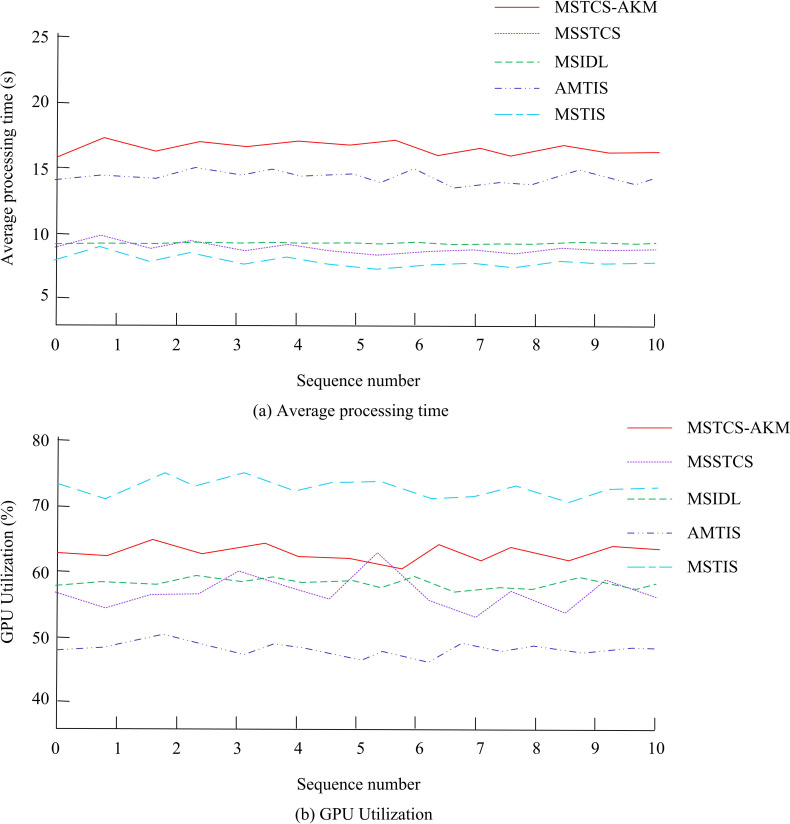
Average processing time versus GPU utilization.

In Fig 12, the average segmentation time of MSTS-AKM was 15 seconds, MSTS-AKM needed to search and calculate multiple possible segmentation points, which resulted in it not performing as well as other algorithms in terms of time efficiency. However, the difference was not significant. Further comparative analysis was conducted on the average processing time and GPU utilization of five algorithms for generating human animations in Fig 13.

In Fig 13 (a), the average processing time for generating human animation in MSTS-AKM was 17 seconds, which was longer than the other four methods, but only 8 seconds behind. The other performance of MSTS-AKM was even better. Overall, MSTS-AKM was still the best. In Fig 13 (b), the average GPU utilization rate of MSTS-AKM was 70%, which had noobvious difference with the other four methods, indicating that these algorithms had similar optimization levels in parallel computing or GPU acceleration.

### 4.3. Comparison of human animation generation effects

Finally, the effects of different methods for generating human animation were compared, and various indicators were normalized in Table 1.

**Table 1:** Comparison of the effects of human animation generated by different methods.

Evaluation index	MSTS-AKM	MSTIS	AMTIS	MSSTCS	MSIDL
Color fidelity	0.91	0.76	0.82	0.74	0.76
Detail presentation	0.93	0.77	0.86	0.75	0.78
Movement fluency	0.89	0.68	0.84	0.71	0.74
Frame to frame continuity	0.92	0.72	0.73	0.77	0.72
Overall animation consistency	0.95	0.74	0.75	0.69	0.79
Action authenticity	0.91	0.83	0.71	0.81	0.82
Role expressiveness	0.95	0.88	0.80	0.73	0.81

**Fig 14:**
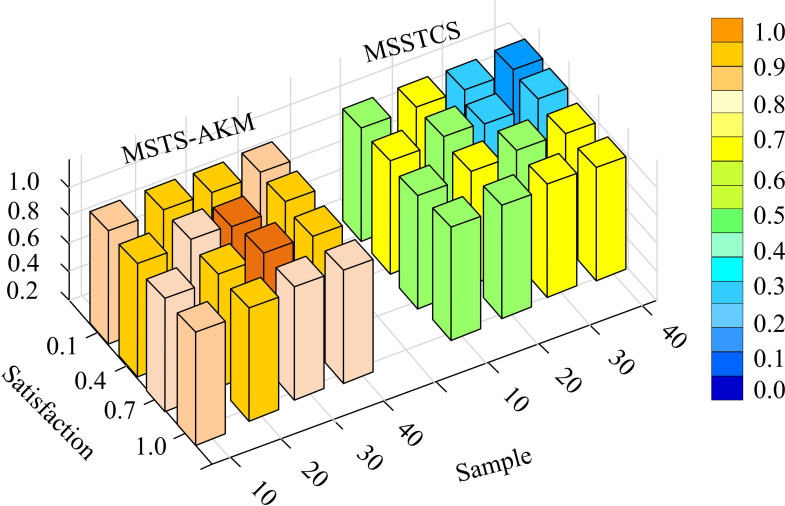
Human animation generation effect comparison.

In Table 1, the color realism, detail representation, action fluency, frame to frame coherence, overall animation consistency, action realism, and character expressiveness of MSTS-AKM generated human animation were 0.91, 0.93, 0.89, 0.92, 0.95, 0.91, and 0.95, respectively, which were higher than other methods. MSTS-AKM demonstrated comprehensive and excellent performance in human animation generation, particularly in terms of color realism, detail representation, overall animation consistency, action realism, and character expressiveness. These advantages made it a powerful tool for generating high-quality human animations. Finally, this study compared the human animation generation effects of MSSTCS and MSTS-AKM based on user satisfaction in Fig 14.

In Fig 14, the average user satisfaction rate for generating human animations using MSTS-AKM was above 0.85. The average user satisfaction rate for generating human animations using MSSTCS was only 0.4. MSTS-AKM in generating human animation had higher user satisfaction than MSSTCS. This is mainly due to its superior performance in visual effects, smooth movements, emotional expression, and other aspects.

## 5. Discussion

The proposed MSTS-AKM algorithm performed well in both coarse-grained and fine-grained segmentation accuracy, which was stable at 0.96 and 0.91, respectively, significantly superior to the other four algorithms. This result verified the strong performance of the algorithm in image segmentation tasks. Especially after the introduction of Gaussian noise with different variances, MSTS-AKM algorithm showed strong robustness to noise, and its accuracy decreased less, compared with other algorithms, its decrease rate was up to 85%. This showed that MSTS-AKM algorithm could maintain high segmentation accuracy when dealing with common noise interference problems in practical applications. The high segmentation accuracy and robustness of MSTS-AKM algorithm were mainly attributed to several key factors. Firstly, the algorithm adopted a tree-like hierarchical model based on the human skeleton topology, which helped to maintain the consistency of bone length in the filtering process. Secondly, the application of bidirectional unbiased Kalman filter improved the accuracy of estimating the rotation angle of the node from noisy data. In addition, based on the application of DASR and MSTCS, the algorithm could automatically identify the key motion segments of behavioral pattern change without manually specifying the number of clusters. Compared with the Action2Video method proposed by Guo et al. [6], MSTS-AKM algorithm performed better in terms of motion fluency and realism. Action2Video, while capable of generating 2D videos from 3D poses, may lack sufficient detail when dealing with complex motion transitions. In contrast, the MSTS-AKM algorithm could more accurately capture subtle changes in motion through a detailed segmentation strategy, resulting in a more natural and realistic animation. Compared with the GANimator model proposed by Li et al. [7], MSTS-AKM algorithm had obvious advantages in segmentation accuracy. GANimator models, while capable of learning and synthesizing new motion patterns from a single motion sequence, could face challenges when dealing with large data sets and complex motions. The MSTS-AKM algorithm overcame this limitation by clustering algorithm based on sparse representation and improved the processing ability of complex actions. Tous et al. ‘s Pictonaut method [8] realizes video cartoonization through 3D human pose estimation and GANs, but its naturalness and diversity of animation may not be as good as MSTS-AKM algorithm. MSTS-AKM algorithm not only improved the naturalness of animation by integrating various algorithms, but also increased the diversity of animation, making it more suitable for different application scenarios. Although the MSTS-AKM algorithm is excellent in many aspects, it still has some limitations. For example, performance when dealing with extreme noise and complex action transitions requires further validation and optimization. In addition, the time efficiency and real-time performance of the algorithm also need to be improved to meet the needs of real-time motion capture and animation generation.

## 6. Conclusion

To facilitate the management of motion data and improve the effectiveness of human animation generation, this study proposed a human animation generation method that integrated motion smoothing algorithms and motion segmentation algorithms. Efficient segmentation and animation generation of action sequences were achieved through bidirectional unbiased Kalman filters and sparse representation-based clustering algorithms. In summary, MSTS-AKM performed excellently in segmentation accuracy, noise resistance, time complexity, utilization, and human animation generation effects. Although slightly lacking in time efficiency, its significant advantages in segmentation accuracy and user satisfaction make it a highly promising algorithm in human animation generation. However, there are still some limitations to the research. The limitations of the current research are mainly reflected in the sensitivity of the algorithm to noise and the performance of the complex action transformation processing. Although the MSTS-AKM algorithm performs well under conventional conditions, the robustness of the algorithm still needs to be enhanced under high noise environments. In addition, when the algorithm deals with fast and complex action transitions, there may be detail loss, which affects the fluency and realism of the animation. Future work will focus on the improvement of the noise filtering capacity of the algorithm and the refinement of the motion conversion. Specifically, more advanced noise suppression techniques will be explored, optimizing algorithms to better capture subtle changes in motion. At the same time, the time complexity of the algorithm is studied, and the real-time performance of the algorithm is improved by parallel computing and hardware acceleration, so that it is more suitable for real-time motion capture and animation generation scenes.
